# The Uptake and Accuracy of Oral Kits for HIV Self-Testing in High HIV Prevalence Setting: A Cross-Sectional Feasibility Study in Blantyre, Malawi

**DOI:** 10.1371/journal.pmed.1001102

**Published:** 2011-10-04

**Authors:** Augustine Talumba Choko, Nicola Desmond, Emily L. Webb, Kondwani Chavula, Sue Napierala-Mavedzenge, Charlotte A. Gaydos, Simon D. Makombe, Treza Chunda, S. Bertel Squire, Neil French, Victor Mwapasa, Elizabeth L. Corbett

**Affiliations:** 1 Malawi-Liverpool Wellcome Trust Clinical Research Programme, Blantyre, Malawi; 2 Liverpool School of Tropical Medicine, Liverpool, United Kingdom; 3 Department of Infectious Disease Epidemiology, London School of Hygiene and Tropical Medicine, London, United Kingdom; 4 Faculty of Epidemiology and Population Health, London School of Hygiene and Tropical Medicine, London, United Kingdom; 5 Division of Infectious Diseases, Department of Medicine, Johns Hopkins University, Baltimore, Maryland, United States of America; 6 HIV Unit, Ministry of Health, Lilongwe, Malawi; 7 Faculty of Infectious and Tropical Diseases, London School of Hygiene and Tropical Medicine, London, United Kingdom; Massachusetts General Hospital, United States of America

## Abstract

**Background:**

Although HIV testing and counseling (HTC) uptake has increased dramatically in Africa, facility-based services are unlikely to ever meet ongoing need to the full. A major constraint in scaling up community and home-based HTC services is the unacceptability of receiving HTC from a provider known personally to prospective clients. We investigated the potential of supervised oral HIV self-testing from this perspective.

**Methods and Findings:**

Adult members of 60 households and 72 members of community peer groups in urban Blantyre, Malawi, were selected using population-weighted random cluster sampling. Participants were offered self-testing plus confirmatory HTC (parallel testing with two rapid finger-prick blood tests), standard HTC alone, or no testing. 283 (95.6%) of 298 selected adults participated, including 136 (48.0%) men. 175 (61.8%) had previously tested (19 known HIV positive), although only 64 (21.5%) within the last year. HIV prevalence was 18.5%. Among 260 (91.9%) who opted to self-test after brief demonstration and illustrated instructions, accuracy was 99.2% (two false negatives). Although 98.5% rated the test “not hard at all to do,” 10.0% made minor procedural errors, and 10.0% required extra help. Most participants indicated willingness to accept self-test kits, but not HTC, from a neighbor (acceptability 94.5% versus 46.8%, *p* = 0.001).

**Conclusions:**

Oral supervised self-testing was highly acceptable and accurate, although minor errors and need for supervisory support were common. This novel option has potential for high uptake at local community level if it can be supervised and safely linked to counseling and care.

*Please see later in the article for the Editors' Summary*

## Abstract 2

Augustine Choko and colleagues assess the uptake and acceptability of home-based supervised oral HIV self-testing in Malawi, demonstrating the feasibility of this approach in a high-prevalence, low-income environment.

## Editors' Summary

**Background:**

According to the World Health Organization, despite the dramatic increase in the acceptability of HIV testing, more than 60% of people living with HIV do not know their status—a factor that is seriously hampering the global response to the HIV epidemic. The inconvenience and cost involved in visiting services in addition to a general aversion to visiting health facilities appear to be major barriers. Home-based HIV-testing services bypass these obstacles and are being adopted as national policy in a number of countries. However, given the tension between confidentiality and convenience, many people do not want to be counseled and tested by someone they know well, thus creating logistical difficulties and added costs to the provision of home-based testing services.

**Why Was This Study Done?:**

Self-testing in private has considerable potential to contribute to first-time and repeat HIV testing but raises a number of issues, such as accuracy, the potential for adverse psychological reactions in the absence of face-to-face counseling, and the difficulty in organizing subsequent links to HIV/AIDS care. Self-testing has been used for over a decade in the US, but given the need to further scale up HIV testing and counseling in Africa, and to encourage regular repeat testing, the researchers conducted a mixed quantitative and qualitative study of self-testing for HIV using oral test kits to test whether supervised oral self-testing could yield accurate results. The researchers also wanted to explore reasons for accepting self-testing and respondents' preferences for HIV testing.

**What Did the Researchers Do and Find?:**

The researchers conducted their study in four community health worker catchment areas in three high-density residential suburbs of Blantyre, Malawi. Between March and July 2010, the researchers randomly selected two groups of participants from within these catchment areas and all adults were then invited to participate in interview and optional HIV testing and counseling carried out in their home. Participants were offered the choice between self-test for HIV followed by standard voluntary counseling and testing, standard voluntary counseling and testing only, and no HIV testing or counseling. Pre-and post-test counseling was provided to all participants and after self-testing, a counselor reread the self-test kit, completed a checklist of potential errors and confirmed the result using two rapid HIV test kits run in parallel from a finger-prick blood specimen. All participants testing positive were referred to the nearest primary health center.

All 260 participants who consented to voluntary counseling and testing also opted to self-test, with the remaining 23 (8.1%) choosing not to test. HIV prevalence was 18.5% (48 of 260) and HIV prevalence among participants who had previously tested HIV-negative or not tested at all was 12.0% (29 of 241 participants) meaning that less than half of HIV-infected participants were previously diagnosed, and just over half of undiagnosed HIV infections were in individuals who had previously tested HIV negative. The researchers found self-testing to be highly accurate, with clear and concordant results for 256 (99.2%) of 258 participants with both self-test and blood results. Overall sensitivity for self-test self-read was 97.9% with specificity of 100%. At exit interview, 256 (98.5%) of participants rated self-testing as “very easy” to do but additional help was requested by 26 (10%) of self test participants and procedural errors were identified for 26 participants (10%). Importantly, self-testing was the preferred option for future HIV tests for 56.4% of participants and the most common choice for both men and women.

**What Do These Findings Mean?:**

The findings of this study show that self-testing for HIV (after a brief demonstration and illustrated instructions) is highly accurate and is widely accepted by the community, indicating that there is strong community readiness to adopt self-testing alongside other HIV counseling and testing strategies in high HIV prevalence settings in urban Africa. Self-testing may prove especially valuable for encouraging regular repeat testing, couple testing, and first-time testing in otherwise hard-to-reach groups such as men and older individuals. Finally, given the accuracy achieved and strong preferences around future testing, further exploration of self-testing options could help to make progress towards meeting universal access goals.

**Additional Information:**

Please access these Web sites via the online version of this summary at http://dx.doi.org/10.1371/journal.pmed.1001102.

- This study is further discussed in a *PLoS Medicine* Perspective by Walensky and Bassett
- Recently published WHO Guidelines explain the principles and processes of adapting HIV guidelines into national programs
- The U.S. Centers for Disease Control's initiative Act against AIDS has some user-friendly information on the different types of HIV tests available
- A WHO document discusses existing practices and surrounding issues related with HIV self-testing among health workers in sub-Saharan Africa

## Introduction

The acceptability of HIV testing has increased dramatically in the last few years in countries with generalized HIV epidemics, as evidenced by increasingly low refusal rates when adults are directly offered convenient HIV testing either in health facilities [1],[2] or through home-based HIV testing [3]–. In Southern Africa, where adult HIV prevalence is far higher than in any other region [8], only a small minority of adults who do not already know their HIV status have no intent to test in future [8],[9]. However, use of facility-based and free-standing voluntary HIV testing and counseling services has remained well below levels of expressed interest and intent, particularly for men, rural populations, and the very poor [6],[8]–[10]. The inconvenience and cost involved in visiting fixed-site services, as well as a general aversion to visiting health facilities, appear to be major deterrents [4]–[6],[8]–[12].

Home-based HIV-testing services bypass these barriers, have much greater acceptability at population level [4],[5],[9],[12], and are being adopted as national policy in a number of countries [7],[8],[13]. Unfortunately, a common finding is that many people do not want to be counseled and tested by someone they know personally, or even access services being used by people they know [5],[7],[12]. This situation creates a strong tension between confidentiality and convenience, and adds considerably to the cost and logistical difficulty of providing home-based services [4],[5],[7],[12],[14].

Self-testing in private has considerable unexplored potential to contribute to first-time and repeat testing for HIV, and could potentially meet the requirement for anonymity at the time of home-testing [7],[11],[15],[16]. Self-testing, however, raises a number of issues, relating to its accuracy, the potential for adverse psychological reactions in the absence of face-to-face counseling [16], and the difficulty in organizing subsequent linkage into HIV/AIDS care [15]–[18]. Reassuring results have been obtained from the US, where home collection of specimens followed by telephoned results and counseling has been available for over a decade [16]: postmarketing surveillance has not identified any increased risk of suicide, and subsequent linkage into care appears similar to that obtained with other testing options [19].

Given the need to further scale up HIV testing and counseling in Africa [8] and encourage regular repeat testing [8], we carried out a mixed quantitative and qualitative study of self-testing for HIV using oral test kits in urban Blantyre, Malawi, with confirmatory blood-based HIV testing and post-test counseling. To the best of our knowledge, this constitutes the first study of the use of oral self-testing among the general population of a country affected by a generalized epidemic. The main aim was to test whether supervised oral self-testing could yield accurate results. We also explored reasons for accepting self-testing and respondents' preferences for HIV testing.

## Methods

### Community and Participant Selection

Population-weighted random cluster sampling, using community-health worker catchment areas as the primary sampling unit, was used to select four community-health worker catchment areas from Ndirande, Likhubula, and Chilomoni high-density residential suburbs of Blantyre, Malawi. Boundaries were defined by capturing Global Positioning System (GPS) coordinates on a circumferential walk. These coordinates were superimposed onto high resolution satellite maps (March 2010 images: Geo Eye-1/Eurimage SpA) using Google Earth Pro software. Between March and July 2010, two groups of participants were randomly selected from within these catchment areas for interview and offer of HIV counseling and testing.

### Adults in Randomly Selected Households

**Figure 1:**
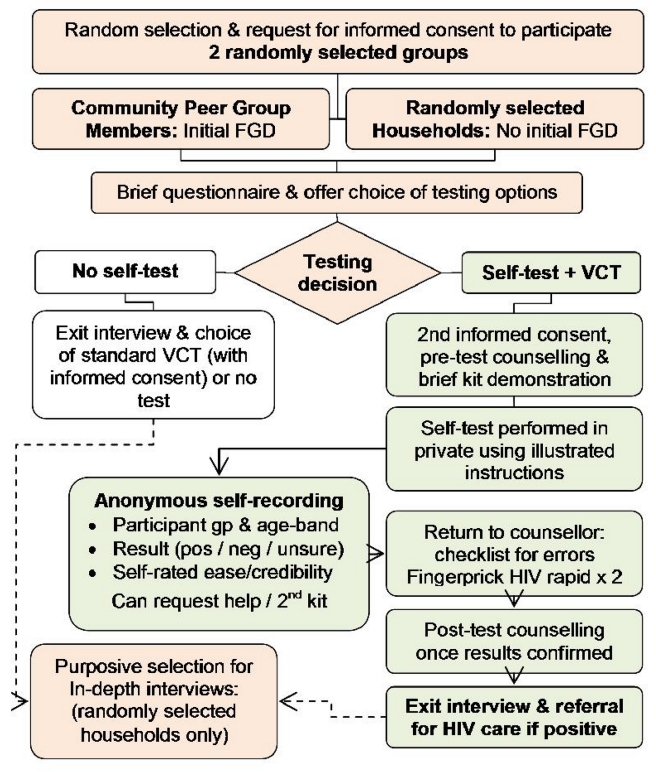
Schematic of study design.

Identity numbers were assigned to all visible dwellings, followed by random selection of 15 dwellings from each selected catchment area. A random list of five further dwellings was available to replace any abandoned or nonresidential buildings. Dwellings were visited to introduce the study, identify all individual households (defined by sharing food), and allow a final random selection of a single household from each dwelling. All adults (≥16 y) were then invited to participate in interview and optional HIV testing and counseling (Figure 1) carried out in the participant's home.

Purposive sampling was then used to select participants for in-depth interviews held within a few days of self-testing, aiming to include up to 36 participants to represent single men, single women, and couples, and to explore choice between the different HIV test options (see below). Interview guides aimed to situate individual experiences of self-testing within broader lifestyle narratives including decision-making, testing preferences and practices, health seeking experiences, and individual level barriers and facilitators.

### Community Peer-Group Members

To facilitate informative focus group discussions, peer groups, such as sports teams, micro-finance, and church groups, that were active in the four selected catchment areas were identified through situational analysis and community mapping exercises. Eligibility was restricted to groups without a health or HIV theme. Six groups were randomly selected for inclusion, followed by random selection of 12 individuals from each group for a 2-h focus group discussion held at a local community venue. These discussions explored community views on barriers and facilitators to self-testing including use amongst couples, the role of supervision and counseling, preferences for different voluntary counseling and testing (VCT) options, and a demonstration and discussion of the self-testing process. All participants were then offered HIV testing and counseling on an individual basis (see Figure 1).

### Optional HIV Testing and Counseling

Consenting adults selected as above were asked to consent to a questionnaire capturing demographic and socioeconomic data, previous HIV test history, and test preferences (with a stigma module also administered to adults from randomly selected households), before being offered the choice between: self-test for HIV followed by standard VCT; standard VCT only; and no HIV testing or counseling.

Participants were asked to consider HIV testing even if they knew themselves to be HIV positive, to allow general population HIV prevalence to be estimated and to maximize power to investigate specificity.

Participants opting to self-test had pretest counseling and a brief demonstration of an oral test kit. They then collected their own specimen without intervention, developed and read the results, guided by illustrated instructions. A brief anonymous self-administered questionnaire captured participant recruitment group, 10-y age band, results, ease, credibility, self-identified errors, and self-test results. This questionnaire was read to illiterate participants. Participants could request additional help if needed. After self-testing, a counselor reread the self-test kit, completed a checklist of potential errors, and confirmed the result using two rapid HIV test kits run in parallel from a finger-prick blood specimen. After post-test counseling and (where indicated) written referral into HIV care services, an exit interview was conducted by a research assistant unaware of the participant's HIV result. The exit questionnaire included a section filled by the counselor on self-test accuracy, and errors made, but not the test result.

### Ethical Considerations

All participants provided written (or witnessed if illiterate) informed consent. Pre- and post-test counseling was provided to all participants. All participants testing positive were referred to the nearest primary health center for HIV care. Approval was obtained from the ethics committees of London School of Hygiene and Tropical Medicine (UK) and College of Medicine Research Ethics Committee, Blantyre, Malawi.

### Laboratory Methods

Self-testing used OraQuick ADVANCE HIV I/II (OraSure Technologies) run from an oral mucosal transudate specimen. Confirmatory testing used Determine (Abbott Laboratories) and Unigold (Trinity Biotech plc), with a third test (SD Bioline HIV I/II Standard Diagnostics, Inc.), together with repeat of all three previously used kits, used in the event of any discordance. Test kits were stored in a temperature-controlled laboratory in compliance with recommended storage between 2 to 27°C, before use at ambient temperatures that were within the manufacturers recommendations (15–37°C) at the time of the study.

### Sample Size

Sample sizes were based around sufficient precision to exclude accuracy of self-testing worse than 95% if our measured estimate was 98%. Our sampling strategy aimed to provide 200 to 250 participants accepting self-testing, on the basis of three adults per household and an 80% or higher uptake of self-testing in community participants.

### Statistical Methods

Quantitative data were entered and managed using Epi info version 3.4.3 (Centers for Disease Control and Prevention, Atlanta, Georgia, US). Analysis used Stata version 10.0 (Stata Corporation).

Questionnaires used mainly close-ended questions with predefined categorical coding, on the basis of previously published and author-supplied questionnaires as far as possible [20]–[26]. Demographic variables were recoded into categorical variables on the basis of frequency of responses before substantive data analysis. Where possible, variables with similar themes were combined. Occupation was classified into six categories, using the nature of the job and security of contract. Twelve questions related to stigma were combined into an ordered categorical scale, having first shown acceptable internal consistency using Cronbach's alpha [27].

95% confidence intervals for the proportion of self-tests accurately read, and for sensitivity and specificity of the tests were calculated using the Agresti-Coull approach [28]. Participants with no confirmatory results were excluded; inconclusive results were treated as inaccurate for sensitivity and specificity analyses. The clustered sampling design was taken into account using the complex survey commands in Stata, which use linearization to estimate standard errors. Categorical baseline characteristics were compared between the two selection groups, and between males and females, using design-based *F*-tests calculated from applying the second order Rao and Scott correction [29],[30] to the usual Pearson chi-squared test statistic for two-way tables. Continuous baseline characteristics were compared between selection groups using logistic regression, adjusted for clustering.

Risk factors were explored for the main outcomes of interest (uptake, accuracy, acceptability, and reported ease of self-testing) using the *F*-test described above for categorical variables and logistic regression, adjusted for clustering, for continuous or ordered categorical variables. Logistic regression, adjusted for clustering was used for multivariate analysis, including variables identified as having a priori association with outcomes of interest, as well as other variables with univariate association at *p*≤0.1. Thereafter a stepwise approach was taken, removing variables with *p*>0.1 from the multivariate model.

Qualitative data were recorded, transcribed, translated, quality checked, imported to NVIVO software for qualitative data analysis version 8.0 (QSR), and coded thematically using a framework developed deductively from the study objectives and reordered inductively from a grounded approach to theme identification. Analysis used a constant comparative approach, identifying patterns in the data, situating these and testing developing hypotheses on all cases, reexamining data contradictions within broader social contexts.

## Results

### Participants

283 (95.6%) of 298 selected individuals consented to interview, including 216 of 226 (95.6%) adults from the 60 randomly selected households, and 67 of 72 (93.1%) community peer-group members (see flow diagram, Figure 1).

Baseline characteristics by selection group are shown in Table 1. 147 (51.9%) of participants were women, with median age of 27 y. Only 30 (10.6%) participants were in regular employment, and regular or recent food shortage in the household was reported by 39 (58.2%) peer-group members and 55 (25.5%) adults from randomly selected households (design-based *F*(1,3) = 15.65, *p* = 0.029). Peer-group members were significantly more likely to report worrying a lot about HIV (27; 41.5%) than adults from randomly selected households (42; 19.4%, design-based *F*(1,3) = 85.69, *p* = 0.003). Otherwise baseline characteristics were similar between selection groups.

**Table 1:** Participant characteristics by selection group.

Characteristic	Peer-Group Member (*n* = 67)	Household Member(*n* = 216)	*p*-Value
**Gender**			
Male	30 (44.8%)	106 (50.9%)	0.532*
Female	37 (55.2%)	110 (49.1%)	—
**Age**			
Median (IQR)	27 (23–32)	26.5 (22–32)	0.302**
**Occupation**			
Regular employment	8 (11.9%)	22 (10.2%)	0.726*
**Marital status**			
Single	20 (29.9%)	66 (30.6%)	0.977*
Married	40 (59.7%)	128 (59.2%)	—
Divorced/widowed	7 (10.4%)	22 (10.2%)	—
**Stigma score**			
Low	ND	94 (44.1%)	ND
Low-medium	ND	52 (24.4%)	—
Medium-high	ND	46 (21.6%)	—
High	ND	21 (9.9%)	—
**Literacy**			
Unable to read	4 (6.0%)	19 (8.8%)	0.611*
**Education**			
Part primary/None	5 (7.5%)	17 (7.9%)	0.176**
Primary or lower	20 (29.9%)	72 (33.3%)	—
Higher	42 (62.7%)	127 (58.8%)	—
**Goes hungry**			
Sometimes	27 (40.9%)	52 (24.1%)	0.029**
Often/always	12 (18.2%)	3 (1.4%)	—
**Previous HIV test**			
Ever tested before	38 (56.7%)	137 (63.4%)	0.372*
**HIV test within last year**			
Yes	16 (23.9%)	48 (22.2%)	0.652*
**HIV Risk: own perception now or future**			
Medium	16 (24.6%)	35 (16.2%)	0.570**
High	19 (29.2%)	79 (36.6%)	—
**Worries a lot about HIV**			
Yes	27 (41.5%)	42 (19.4%)	0.003*
**Knows AIDS death personally**			
Yes	50 (74.6%)	171 (79.2%)	0.657*
**Testing choice**			
Self-test + VCT	62 (92.5%)	198 (91.7%)	0.842*
No test	5 (7.5%)	18 (8.3%)	—

*p-Value from *F*-test (categorical variables).

**p-Value from logistic regression (ordered categorical/continuous variables) adjusted for clustered design.

ND, not determined.

Approximately two-thirds of the women (118, 67.4%), but only one-third (55, 32.6%) of the men had previously tested for HIV (design-based *F*(1,3) = 56.20, *p* = 0.005). Recent HIV testing (within the last year) was reported by 47 (32.0%) women and 17 (12.5%) men (design-based *F*(1,3) = 18.01, *p* = 0.024).

### Uptake of Offer of Self-Testing and HIV Prevalence

Choices made between the three testing options (no test, VCT only, self-test plus VCT) are shown in Table 1. All 260 (91.9%) participants who consented to VCT also opted to self-test, with the remaining 23 (8.1%) choosing not to self-test. Differences between those accepting and declining self-testing were minor, with significant differences being limited to a higher proportion of those who reported to be worried a lot about having HIV (design-based *F*(1,3) = 14.44, *p* = 0.016).

Two participants with missing confirmatory results (one opt out, one incomplete form) were not included in accuracy analyses. HIV prevalence was 18.5% (48 of 260), with positive results in 37 (18.8%) of 197 randomly selected community adults, and 11 (17.5%) of 63 community peer-group members. HIV prevalence among participants who had previously tested HIV-negative or not tested at all was 12.0% (29 of 241 participants).

### Accuracy of Self-read Self-testing Compared to Confirmatory Blood Testing

Self-testing was highly accurate, with clear and concordant results for 256 (99.2%; 95% CI 97.0–100.0%) of 258 participants with both self-test and blood results (Table 2). One HIV-positive participant was unable to interpret a faint positive test (recording the result as “uncertain”), and one HIV positive participant had no discernible line within the recommended test period. In both cases a repeat test performed by the counselor gave the same findings.

**Table 2:** Cross tabulation of OraQuick and confirmatory blood test results in all participants and participants not already known to be HIV positive.

OraQuick Self-test	Positive	Negative	Inconclusive	Unconfirmed	Total
***All participants***					
Positive	46	0	0	0	46
Negative	1	210	0	2	213
Inconclusive	1	0	0	0	1
Total	48	210	0	2	260
***Excluding HIV-positive participants who have tested positive before***					
Positive	27	0	0	0	27
Negative	1	210	0	2	213
Inconclusive	1	0	0	0	1
Total	29	210	0	2	241

Participants with no confirmatory results are not included in sensitivity and specificity analyses. Inconclusive results are considered to be inaccurate (false) results in sensitivity and specificity analyses.

**Figure 2:**
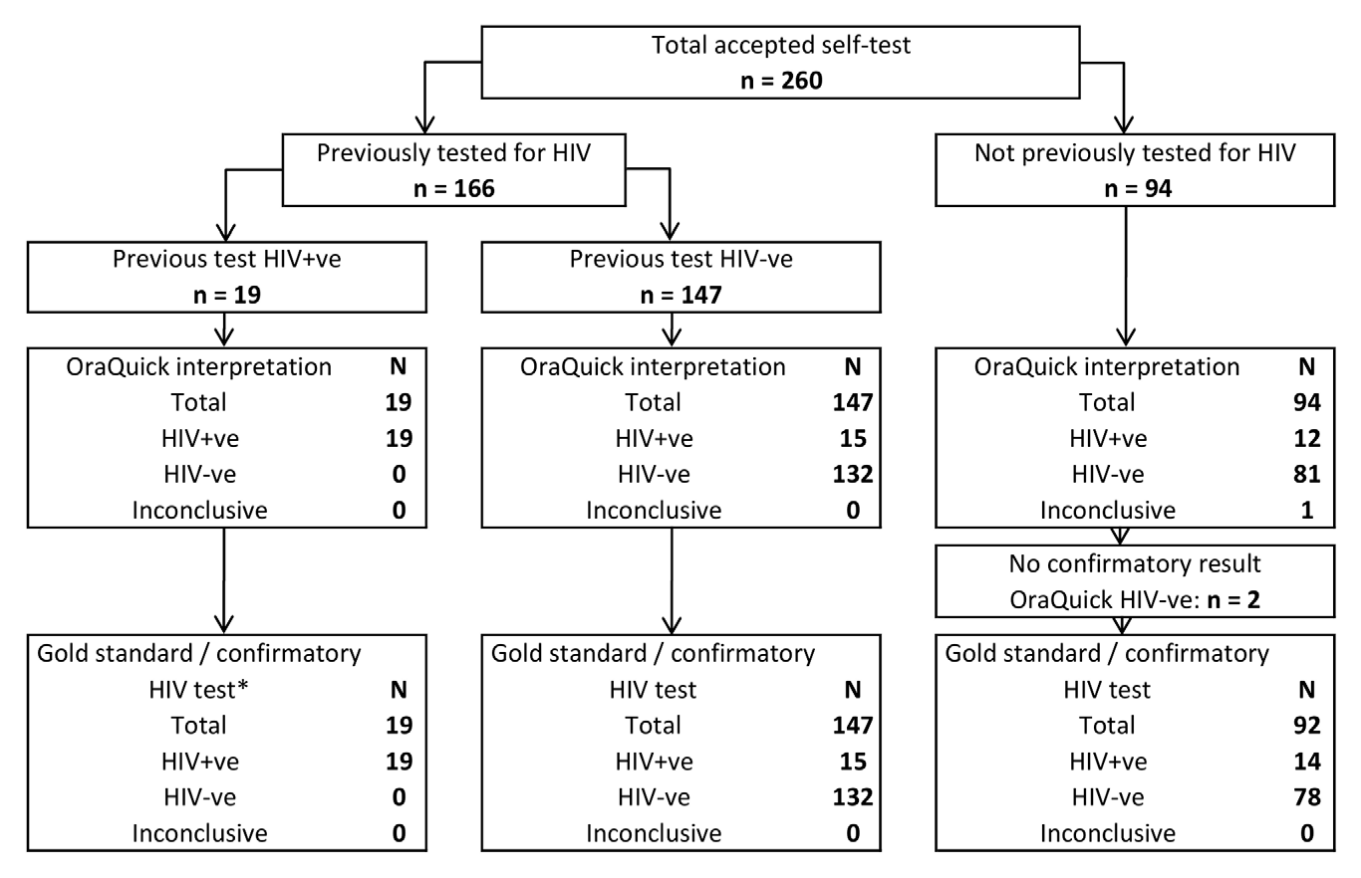
Accuracy of self-test self-read results against gold standard. Parallel testing of finger-prick blood by a counselor using Determine and Unigold, Bioline if discordant.

Overall sensitivity for self-test self-read was 97.9% (95% CI 87.9%–100.0%) with specificity of 100% (95% CI 97.8%–100%), respectively (Table 2). Results according to previous test results (positive, negative, or no previous test) are summarized in Figure 2. Table 2 shows the cross-tabulation of self-test and confirmatory results in 241 participants not previously known to be HIV positive. In this subgroup sensitivity was 96.4% (81.7%–99.9%) with specificity 100% (98.3%–100.0%).

### Reported Ease of Self-testing, Need for Help and Errors

At exit interview, 256 (98.5%) of participants rated self-testing as “very easy” to do. The visual and verbal demonstration of kit use was considered as useful as the illustrated instructions provided by the counselor when carrying out self-testing (250/260, 96.2% and 252/260, 96.9% respectively).

Additional help beyond a brief demonstration and illustrated instructions was requested by 26 (10.0%) of self-test participants (Table 3), most commonly to clarify taking the mouth swab. Illiterate individuals (6/22, 27.3% compared to 15/236 literate, 6.4%; design-adjusted *F*(1,3) = 7.51, *p* = 0.071), those with lower levels of schooling (design-based *F*(1,3) = 7.93, *p* = 0.067) and women (15/134, 11.2% compared to 6/124, 4.8% men, design-adjusted *F*(1,3) = 12.14, *p* = 0.040) requested significantly more assistance. The nature of help requested is summarized in Table 3.

**Table 3:** Help required and errors made by participants when self-testing.

Category	*n*	Percent
**Needed help**		
Asked counselor to watch and confirm correct approach to taking mouth swab	8	3.1
Asked for clarification after making a minor error	6	2.3
Asked for help opening developer fluid vial	4	1.5
Unable to use timer	2	0.8
Unable to take own mouth swab (asked counselor to do for them)	2	0.8
Unable to read result (asked counselor to read with them)	2	0.8
Wanted counselor present during collection	2	0.8
***Total***	**26**	**10.0**
**Errors made**		
Incorrect or incomplete swab of upper and lower gums	4	1.5
Touched flat pad	15	5.8
Spilt developer fluid	2	0.8
Fumbled vial or cap when opening developer fluid	1	0.4
Removed kit from developer too early	3	1.2
Read incorrectly (faint positive read as negative)	1	0.4
***Total***	**26**	**10.0**
Required second kit because of inconclusive result	2	0.8

Procedural errors were identified for 26 participants (10.0%, Table 3). The most serious errors, with highest potential to affect the results were: removing kit from developer too early (three participants, 1.2%) and spilling the developer fluid (two participants, 0.8%). Two participants (0.8%) required a second kit because of inability to read a very faint test line on a self-test result.

### Acceptability of Self-testing in the General Community

Acceptability was assessed both before and after self-testing, with results summarized for adults from randomly selected households in Table 4. As detailed in the Methods, self-testing options were ranked second to door-to-door standard VCT by an external provider as being most likely to successfully increase HIV testing in the community (Table 4). Local provision of standard VCT by a neighbor was unacceptable to most women 71 (64.6%) and a substantial minority of men 41 (38.7%, design-based *F*(1,3) = 13.13, *p* = 0.036), whereas local distribution of self-test kits by a neighbor without having to disclose results was acceptable to 205 (94.5%) of participants with no differences between males and females (design-based *F*(1,3) = 0.03, *p* = 0.878).

**Table 4:** Preferences and acceptability of HIV testing and counseling options.

Adults from Randomly Selected Households in Blantyre	*n* = 110 Women (%)	*n* = 106 Men (%)	*p*-Value* (Gender)
**Before self-testing**			
*Willingness to test if HIV services were provided by a neighbor?*	—	—	0.036
Willing to self-test (do not have to reveal result)	66 (60.0)	38 (35.9)	—
Willing to accept both self-testing and standard VCT	38 (34.6)	63 (59.4)	—
Not willing to accept either self-testing or standard VCT	6 (5.5)	5 (4.7)	—
*Strategy most likely to be successful in increasing testing in the community*			
Door-to-door testing by counselor from outside community	59 (53.6)	50 (47.2)	0.737
Door-to-door testing with local person as counselor	11 (10.0)	15 (14.2)	—
Mobile clinics providing VCT	12 (11.0)	10 (9.4)	—
Free self-test kits available from local vendors and stores	14 (12.7)	6 (5.6)	—
Door-to-door self-testing provided by local person	14 (12.7)	25 (23.6)	—
Either of above self-testing option as most/next-most successful	68 (61.8)	76 (71.7)	—
*Agree strongly/somewhat “these days if people have tested before”*			
No need for counseling	12 (10.9)	10 (9.4)	0.378
Telephone hotline sufficient	32 (29.1)	28 (26.4)	0.282
Information leaflet	42 (38.2)	33 (31.1)	0.323
Community worker available	54 (49.1)	53 (50.0)	0.67
**At exit interview**			
Would recommend this kit for self-testing to friends and family^a^	100 (100)	97 (100)	ND
If testing for HIV in future would want next HIV test to be	—	—	0.078
In a VCT centre	3 (2.7)	12 (11.4)	—
At a hospital or clinic	25 (22.7)	13 (12.4)	—
Provided by a counselor at home (door-to-door service)	16 (14.6)	11 (10.5)	—
Self-testing at home	64 (58.2)	68 (64.8)	—
Testing campaign in community (mobile clinic/stand)	2 (1.8)	1 (1.0)	—

a Question only asked to those who accepted self-testing (100 women and 97 men).

*F-test adjusted for the clustered design.

ND, not determined.

After self-testing, all participants would recommend self-testing to friends and family. Self-testing at home was the preferred option for future HIV tests among both men and women (Table 4) and did not significantly vary by age, marital status, schooling, or socioeconomic status (unpublished data).

### Counseling

Despite the popularity of the concept of self-testing, all but a small minority of individuals (22/216, 10.2%) disagreed that people “these days know enough about HIV to do without counseling for a repeat HIV test.” Alternatives to face-to-face counseling for repeat testers, such as telephone helpline, information leaflets, or availability of a local community health worker were also not considered acceptable substitutes by most participants (Table 4).

## Discussion

The main findings of this study of self-testing for HIV are that high levels of accuracy (99.2%) were obtained from oral self-testing after a brief demonstration and illustrated instructions, and that there were strong indicators of community readiness to adopt self-testing alongside other HIV counseling and testing strategies in an African urban high HIV prevalence setting. Self-testing was the preferred option for future HIV tests for 56.4% of participants, being the most common choice for both men and women. There was no obvious subgroup for whom self-testing was not acceptable. These findings are strengthened by our having used random selection of participants from the main high-density residential areas in urban Blantyre, and by the 95.6% participation rate. High-density areas are particularly interesting for self-testing because of the difficulty in securing confidential space for HTC due to lack of soundproofing and intense crowding. The high accuracy contrasts with much lower levels obtained from blood-based self-testing with a less simplified test kit in Singapore [31], but is in the same range as reported for oral self-testing in Americans attending public hospital services [24],[32].

Also remarkable is that 91.9% of individuals who agreed to be interviewed opted to test for HIV, all choosing self-test plus VCT. This finding concurs with results from other recently conducted community-based studies [4],[6] and indicates that knowledge of HIV status has now become sufficiently desirable in many parts of rural and urban Africa to make universal knowledge of recent HIV status by all adults a realistic goal. Given the accuracy achieved and strong preferences around future testing, we consider our results to be sufficiently promising to warrant further exploration of self-testing options as a potential way to make progress towards meeting Universal Access goals [8].

The need for innovation around community-based testing is underscored by the identification of previously undiagnosed HIV in 29 (12.0%) of our participants in this current study. Despite the highly effective public health approach to HIV testing and care taken in Malawi, which is among the most successful programmers in Africa and considered a model for the continent [33],[34], less than half of HIV-infected participants were previously diagnosed, and just over half of undiagnosed HIV infections were in individuals who had previously tested HIV negative. Self-testing may prove especially valuable for encouraging regular repeat-testing, couple testing, and first-time testing in otherwise hard-to-reach groups such as men and older individuals [5],[8],[14],[15].

Other investigators have shown high acceptability, uptake, and high accuracy of oral self-testing in American populations [24],[32], and home-collection of specimens for laboratory HIV testing and telephone results and counseling is well established in America and also available in Europe [15],[16],[18]. Kenya was the first country in Africa to develop policy guidelines for self-testing, although no kit has yet been fully registered [13]. The manufacturers of the kit used in this study have applied to the US Food and Drug Administration (FDA) for registration as an over-the-counter test, and it is likely that self-testing options will become available in many different countries over the next few years.

The high uptake and high proportion of participants selecting self-testing at home as the preferred “next test” option in this current study suggest that carefully considered promotion of self-testing has potential to contribute greatly to scale-up of HIV testing in Africa. For example HIV prevention through “universal test-and-treat” [23]–[26],[35],[36] strategies are being considered with the goal of reducing HIV transmission by prompt identification and initiation of antiretroviral treatment in HIV-infected individuals irrespective of extent of immunosuppression. To be effective such strategies require a high proportion of HIV-infected individuals in communities to be diagnosed and started on treatment within 12 mo of infection, calling for highly acceptable and convenient initial and repeat HIV testing strategies.

The high uptake of home-based testing for HIV and much higher rates of couple testing than alternative strategies, make it the ideal community arm to complement facility-based routine provider-initiated testing in countries with generalized HIV epidemic [3]–[7]. However, sustainability is a key issue, which is compounded by the requirement for counselors who come from outside the community [4],. Home-based self-testing provides privacy from neighbors and may allow development of novel local community strategies: although most of our participants were not willing to accept VCT from a neighbor, 94.9% indicated willingness to accept a self-test kit from a neighbor if they did not have to confide the results. Support is still needed at the time of a positive test, with most participants in the current study considering telephone hot line or information leaflets to be inadequate replacements for face-to-face counseling. Operational research will be needed to investigate whether or not linkage into HIV care is any worse after self-testing than it is for current facility-based HIV testing and counseling services in Africa (estimated linkage into care of less than 50% within 1 y), and ways of providing psychological support following a positive self-test. Examples of potential models include 24-h telephone hotlines, or community workers trained in pretest counseling, distribution of kits, and available for face-to-face post-test counseling. The private nature of self-testing, low potential for inadvertent breach of confidentiality, and ease of incorporating self-testing into busy daily lives, particularly for couple testing, should ideally be retained.

Limitations of the study include the lack of data on linkage into care following a positive HIV test. This area needs to be investigated in any program implementing self-testing, as risk of drop-out between testing HIV positive and entering HIV care is known to be high even under standard VCT models [37]. Linkage into care was not investigated in this study because self-testing was offered in a supervised context, as a first step, so that HIV counselors were able to immediately refer participants into care. Our data on acceptability and accuracy include 19 (7.3% of 260) participants who already know themselves to be HIV-positive and so may have been more willing to test and able to read positive test results accurately. Regulatory strengthening may be required to prevent sales of counterfeit and substandard test kits (as for example has been described in TB diagnostics) [38]. The need to avoid storage over 27°C may be a practical barrier to scale up in hotter climates, although this same recommendation applies to most rapid HIV test kits currently in programmatic use.

In conclusion, we have shown high willingness to self-test for HIV at home when self-testing was linked to confirmatory blood tests, with self-testing the preferred option for future tests. Highly accurate results were obtained by randomly selected African adults in a poor urban setting following a brief demonstration and illustrated instructions. Africans have been living with the spectra of HIV for two decades, bringing personal loss, hardship, and a more uncertain future to almost everyone in the high HIV prevalence Southern region [8],[9],[12]. With the scale up of HIV care, home-based testing and counseling has become a welcome intervention with very high uptake rates [4]–[7],[9]. In settings where regular annual visits to every home by external VCT providers are not feasible, options based on self-testing may offer a more readily sustainable approach that can contribute towards Universal Access goals, provided that mechanisms can be identified to ensure that safety, accuracy, and post-test support are not unduly compromised.

## Abbreviations

HTC: HIV testing and counseling
VCT: voluntary counseling and testing
